# Of Suicide, Illustrated by Anatomico-Pathological Observations

**Published:** 1838-04

**Authors:** 


### Art. IV.

1. *De Suicidio, Observationibus anatomico-pathologicis illustrato.* Scripsit D. J. A. Arntzenius, Med. et Chir. Doct.—*Trajecti ad Rhennon*, 1835. 8vo. pp. 204.

*Of Suicide, illustrated by anatomico-pathological Observations.* By D. J. A. Arntzenius, m.d.

2. *Das Heimweh und der Selbstmord.* Von J. H. G. Schlegel, m.d. &c.—*Hildburghausen*, 1835. I. II. Theil. 8vo. pp. 125, 427.
*Nostalgia and Suicide.* By Dr. Schlegel.

Nothing but the frequency of the fact could make it credible that a rational animal, short-lived and fond of life, should be distinguished, among other things, from all other animals not considered to be endowed with reason, by inflicting premature death upon itself. The circumstances under which the act is done are not calculated to lessen the surprise it occasions. It is resorted to by the young, who might be supposed to be in the enjoyment of the gift of life, and by the old, who might be expected soon to be delivered from it in a natural way. Very often it is performed with evident deliberation and forethought, with much ingenuity of concealment and elaborateness of preparation; and the last act is as calmly executed as if it were but the dignified end of a well-spent life. Charity towards human beings disposes juries, in defiance of barbarous remnants of ancient laws, to assume that, in every case of this kind, the guiding reason was first overthrown; and we believe that this merciful view is actually borne out by the uncoloured facts disclosed by dissection, and facts related by various witnesses, many of whom had had extensive opportunities of observation. The question is, at least, one of considerable interest to every man of humanity, as well as to every man of science. It may be useful to bring together some of the scattered facts bearing upon it, and it may not be uninteresting to the reader to add to these others in various ways connected with the subject of suicide; and for which the two volumes before us afford considerable opportunity.

The little work, to which the name of Dr. Arntzenius is attached, is an enlargement of an academical thesis written by him in 1829, and treats, in a few well-arranged chapters, of suicide in general, of the organic lesions found in suicides, and of the seat and nature of the propensity.

The German book is chiefly a collection of histories of suicides, taken by its author from every available source. Many of the cases have fallen under Dr. Schlegel's own notice: the Bible, old histories, medical and legal records, personal communications, have been the other sources whence much of his matter has been derived; and he has not even neglected to examine newspapers, where, no doubt, his credulity found ample indulgence in reading of "forty naked female corpses, which were, within a period of two days, taken out of the basin in Hyde-Park." But, as we do not regard the whole of his book as of equal authority with the sentence above quoted, we shall notice such parts of it as appear to be most worthy.

After devoting some pages to the Bibliography of Suicide, and some to the various definitions of insanity in which it has been included, Dr. Arntzenius classes it under the head of Delirium sine febre, in a form in which there are false ideas and erroneous judgment concerning one object or a small series of objects; a monomania, in short, according to the term adopted by Esquirol in preference to the term melancholia, which conveys an erroneous impression respecting its origin. Dr. A. then divides suicide into acute and chronic; the first characterized by great physical and moral excitement; the second preceded by sadness, moroseness, fear, and love of solitude; and of the symptoms of the latter, as regards the face, eyes, tongue, respiration, a regular account is given, on which it is impossible to place any reliance. The mode of suicide, it is observed, is that which the unhappy person deems the speediest and the least painful. This, however, is subject to exceptions, many of which the German author has collected. Lord S., in 1834, cast himself into the crater of Vesuvius; a Frenchman, ambitious of leaving the world in a distinguished manner, attached himself to an enormous rocket, to which he then set fire, and thus ended his life; a German, in 1834, cast himself into a smelting furnace. There are, besides, several instances of suicide by abstinence from all nourishment. The American Journal of the Medical Sciences, for August, 1830, relates the case of a young man, aged twenty-seven, who, in a state of religious melancholy, resolved on thus terminating his life. He took nothing but water for seven weeks and four days, at the end of which time he died. An Italian, named Viterbi, according to his own statement, unjustly condemned as an accomplice in a murder which was committed, adopted a similar mode of suicide, and died after twenty-one days; during which time, although food and drink were constantly placed before him, he took only one draught of water and a spoonful of wine. And Dr. Froriep, in his "Notizen," relates another instance of similar self-murder; the object of the suicide being thus to reconcile himself to God. In Paris, the object of terminating existence with as little pain as possible seems to be most cultivated: hence the very frequent use of burning charcoal. And on this account, also, the number of attempted but unsuccessful suicides is far larger in that city than elsewhere; since the poisonous effect of the carbonic acid gas is not unfrequently limited to stupefaction, during which the individual is discovered. The frequency of suicide in Paris, and the trifling circumstances under which many of them originate, have lately been alluded to in the Quarterly Review. Dr. Schlegel has collected some cases of a similar character: one of these is the case of Victor Escousse, a writer of plays. The second of his performances of this kind was blamed by the Parisian journalists: his vanity was mortally wounded, and he killed himself. The suicide of a French cook, because he was disappointed of some fish which was to have supplied a regal dinner, is generally known. An anecdote is here given of a boy, aged thirteen, who put an end to himself, because, as a punishment for some trifling fault, he was not allowed to wear his Sunday's clothes. A German student of law shot himself, from a melancholy state of mind into which he was brought in consequence of a deformity of the foot. The various and irrational shades of lovers' quarrels terminate frequently in the suicide of one or both of the infatuated. Simple ennui appears to be a sufficient inducement to some to end their days. Such an instance is mentioned of two young men, at Versailles, in 1834; and similar instances are not uncommon. The elder was not twenty-four years of age. They appeared possessed of all which could render life agreeable, and were on the most familiar terms with one another. They gradually collected a quantity of charcoal, and, when it appeared that there was but little chance of their being detected until such time as they were no longer in existence, they ignited it, and lay exposed to its fumes. The written excuse for this French folly was "aversion and weariness of life;" the value of which, probably, as far as it concerned their relations to this world, they had properly estimated, by being anxious for its termination. From the following table, it appears that different means of suicide are adopted at different ages:

| | By Pistol. | By Hanging. |
|---|---|---|
| Between 10 years and 20 years of age | 61 | 68 |
| 20 .. 30 ...... | 283 | 51 |
| 30 .. 40 ...... | 182 | 94 |
| 40 .. 50 ...... | 150 | 188 |
| 50 .. 60 ...... | 161 | 256 |
| 60 .. 70 ...... | 126 | 235 |
| 70 .. 80 ...... | 35 | 108 |
| 80 .. 90 ...... | 2 | 0 |
| | 1000 | 1000 |

So that, in the earliest suicidal years, there appears to be a preference for hanging, which afterwards yields to the use of fire-arms; and this is, in more advanced years, again substituted by hanging. In classing 9000 cases of suicide which happened in Paris, between the years 1796 and 1830, Dr. Schlegel concludes that what he terms the "philosophic suicide," or that which is performed after deliberation, is executed during the night, or shortly before sunrise; whilst that which is not the result of premeditation occurs during the day, when its causes are especially operative; e. g. quarrels, mournful intelligence, loss at gaming, intemperance, &c. Drunkenness, says Dr. Schlegel, is the chief cause of suicide in England, Germany, and Russia; love and gambling, in France; whilst bigotry, or the fear of dying without the sacrament, he supposes to prevent it in Spain. In Italy it is very rare. In confirmation of the general opinion respecting its rarity in Italy is given the remark of a Roman lady, on being told that a young man had designedly shot himself: "Dev 'essere un Forestiere; gli Italiani non sono tanto matti." The suicide was a melancholy German tailor.

Suicide, since the revolution in France, has greatly increased. The average number during the last forty-two years has been 409$\frac{5}{6}$. To these must be added the instances in which the attempt on life fails; a circumstance which has been already mentioned as of frequent occurrence; and then, according to the statement of a commission instituted for the purpose of enquiry into this subject, the number must be stated as 1639 annually in Paris; almost as many as in London, taking into consideration the different population of the two cities. Dr. Schlegel alludes to the abandoned state of some of the inhabitants of Paris, when there existed a brotherhood, termed "The Society of the Friends of Suicide." It consisted of twelve members. A lot was annually cast to decide which of them should commit suicide in the presence of the others. Each member of this insane union (proving to what baseness the absence of religion and morals may conduct mankind) was required, by its statutes, (1) to be a man of honour; (2) to have experienced the injustice of mankind, the ingratitude of a friend, or the falseness of a lover or wife; (3) to have experienced, for years, a certain invincible vacuity of the soul, and a discontent with everything in this lower world: "Das Herz ist gestorben —die Welt ist leer." Dr. Schlegel has but little charity towards the French capital. He calls it "a suffocating, boiling cauldron, in which, as in the stew of Macbeth's witches, there simmer, with a modicum of virtue, all kinds of passions, vices, and crimes!"

He has inserted statistical tables of the proportion of suicides to various populations: the first shewing the proportion as it respects the entire country, the second in relation to the inhabitants of cities or large towns. From these we shall make some selections.

FIRST TABLE.

| Countries. | Proportion of Suicides to the Population. |
|---|---|
| 1. Kingdom of Sweden | 1 in 92,375 |
| 2. The Milanese | 1 .. 72,570 |
| 3. Russia, 1819-1820 | 1 .. 36,860 |
| Do. 1824-1827 | 1 .. 34,246 |
| 4. Kingdom of Prussia | 1 .. 14,224 |
| 5. Saxony | 1 .. 8,446. |

Sweden exhibits a small number of suicides; but, when the simple habits of the Swedes, its few towns, and the scattered inhabitants of the country, are considered, the proportion is still considerable.

The estimate for the Milanese relates to the years from 1817 to 1826 inclusive, and is thus divided:

| | | | Individuals of Population. |
|---|---|---|---|
| 1. | Circle of Lodi | yearly, | 1 in 19,410 |
| 2. | Milan (excluding the town) | | 1 .. 35,217 |
| 3. | Cremona | | 1 .. 72,747 |
| 4. | Mantua | | 1 .. 79,082 |
| 5. | Bergamo | | 1 .. 82,012 |
| 6. | Pavia | | 1 .. 91,084 |
| 7. | Brescia | | 1 .. 100,256 |
| 8. | Como | | 1 .. 100,749. |

The numerical proportion of suicides is remarkably different in Russia, according to the governments. In some places, and indeed where the population is the thickest, the proportion is not more than 1 in 100,000; in others it amounts to 8 in 20,000: in this last is included the Siberian governments.

The proportion of suicides is very different in the different provinces of Prussia. It is greatest in that of Brandenberg, if the city of Berlin is included; then follow Saxony, Pomerania, Silesia, and lastly Westphalia and the country bordering the Rhine. In the above classification, we find the greatest number of suicides in Germany, and especially in the territory of the Elbe and Oder. Russia is in this respect intermediate, as it is also with regard to culture and civilization. The fewest suicides are in the most populous and most fertile, and in the least fertile, countries of Europe,—i. e. in Lombardy and Sweden.

SECOND TABLE.

| | Cities. | Proportion of Suicides to the Population. |
|---|---|---|
| Russia | St. Petersburg | 1 in 416 |
| England | London, in the 18th century | 1 .. 10,572 |
| | .... 19th .... | 1 .. 21,491 |
| France | Paris, Department of the Seine, | 1 .. 2,215 |
| Switzerland | Geneva | 1 .. 3,714 |
| Germany | Berlin, 1788-1797 | 1 .. 23,066 |
| | .... 1798-1807 | 1 .. 12,917 |
| | .... 1813-1822 | 1 .. 3,312 |
| | Hamburg | 1 .. 4,800 |
| | Leipzig | 1 .. 3,143 |
| Italy | Milan | 1 .. 18,021 |
| | Naples | 1 .. 27,230 |
| United States of North America | New York | 1 .. 9,474 |
| | Baltimore | 1 .. 15,696 |
| | Philadelphia | 1 .. 20,000. |

Dr. Schlegel appears to think that the estimate of suicides in St. Petersburg, although taken from the *Bulletin Universel*, (sect. 6, tab. 18,) is somewhat too high: in this we cannot but agree with him, or, rather, we think it *much* too high. He thinks also that the diminution in London, during the nineteenth century, is scarcely credible, particularly as the population has so considerably increased. He calls attention to the remarkable increase of suicides in Berlin during successive periods. The Italian towns, as Italy generally, afford but few instances of self-murder. America, in respect to the subject of suicide, does not appear to differ much from the European states.

Self-murder, and other kinds of death, appear to possess this remarkable similarity, that, in a certain period of years, a certain average proportion of each occur. Should not this fact prove that neither causes of a general character, nor changes in the condition of states, occasion or augment suicides; but, rather, that they take their origin in private life? Causes acting generally are of rare occurrence, and do not recur periodically: if to these were to be attributed suicides, they would be occasionally very abundant, but in quiet times very rare, which is contrary to experience. The effects of human passions, exhibiting themselves in private life, do not occur suddenly, like an epidemic, but gradually and in a certain proportion; and these are as little liable to deviation from a certain course as the character and individuality of mankind from whence these passions arise, and produce their effects. But, as the excitement and unrest produced by general causes continue long after their commencement, and are liable to increase from new political events, the increase of suicide may be a *mediate* effect of extraordinary general circumstances.

Among the predisposing causes is enumerated, by Dr. Arntzenius, hereditary disposition. Fabrat has observed families of suicides. The proportion of suicides in men to women is stated to be as three to one, and the majority of suicides in women are occasioned by love. It is most frequent in men and women between the age of puberty and fifty, but there are infantile and senile exceptions. Thus Dr. Schlegel states, on the authority of Casper, that, in Berlin, between the years 1812 and 1821, no less than thirty-one children, of twelve years of age and under, committed suicide, either because they were tired of existence or had suffered some trifling chastisement. The melancholic temperament is, of course, accused; so also a sedentary life, &c.; but this regular detail of causes is fitter for an academical essay than for a practical work; for the student is expected to shew his reading, but the practical reader looks for guidance.

There is nothing in the German work on the morbid appearances found after suicide which requires notice. For these we must refer to the Latin essay.

Without expecting to find anything very satisfactory on this point, it is interesting to see what those who have at least been diligent seekers of information have supposed themselves to discover. But their contrary evidence takes away all feeling of satisfaction. It would, for instance, be interesting, and perhaps instructive, to learn that great thickness of the skull had often been noticed, if it were not accompanied by a precisely similar remark concerning its thinness. The testimony of Greding and Gall is in favour of its frequent thickness; but even this applies to lunatics in general; out of 216 of whom, thickness of the cranium was found in 167. Out of 100 who died with furious mania, 78 had the cranium very thick, and 20 very thin. Out of 30 fatuous patients, 21 had thick crania, and 6 thin. The thickness of the cranial bones in melancholy and maniacal patients, and in old people, was supposed by

Dr. Gall to be connected with a diminished size of the brain, to which the inner table of the cranial bones accommodated itself; and, together with this thickness of the cranial bones, he considered there was thickening of the membranes and ossification of the blood-vessels: but Esquirol denies the existence of thickened cranial bones in the majority of suicides. Dumas, and Baillie, and Everard Home, (quoted as two persons, "Everard *et* Home,") are mentioned as considering thickening of the cranium productive of mental disorder, by compressing the brain. Osiander regards it as an effect of increased vascular action, or a kind of chronic inflammation; and observes, that the same thing is observed in drunkards. In some cases the brain has been found of a capacity smaller than the cavity of the cranium, and in others the capacity of the cranial bones has been considered too small for the brain, and the cause of dreadful cephalalgia or melancholy. Malformation of the cranium, productive of partial pressure, as of the medulla oblongata, by an oblique position of the occipital foramen, or of any portion of the cerebrum, by a depression of the cranial bones, may be a cause of suicide, or of general insanity. More frequently morbid growths at the base of the cranium have been supposed to have such effects. The case of an old man is related by Osiander, who had been long affected with dreadful headach, and at length, weary of life, hanged himself; in whom small osseous excrescences were found near the carotid foramen. In a case mentioned by Lancisi, of hypochondriasis and suicide, a sharp bony excrescence was found near the apex of the lambdoid suture; the whole cranium was in this instance thinner than usual, and diseased from rickets in early life. Cases, similar as respects bony growths and their supposed effects, are quoted from Isenflamm, Elgenstjerna, and Recamier.

Accretions of the membranes to the cranium, or to one another, or to the brain, are also enumerated as being frequently found in suicides. According to Falret, the dura mater not unfrequently presents ossifications, the arachnoid is thickened, and the pia mater inflamed. Vascularity of the dura mater, and a collection of fluid in the ventricles, are mentioned by Home. Osiander considers congestion in the brain as a powerful cause of suicide, and thinks that this often occurs in botanists, whose heads are frequently directed towards the earth, and who "often commit suicide!" But Dr. Arntzenius very properly observes, that the appearances of congestion after death are not always to be depended upon. He quotes Schallgruber, as asserting that effusions between the membranes, sero-sanguineous, milky, albumino-purulent, or bloody, are found in suicides; effects often shewn by no symptoms, until apoplexy or mental disturbance, and fatal consequences, ensue.

Every variety of morbid appearance which has been found in the brain of lunatics, has, as might be expected, been found in suicides: softening, hardening, toughness, abscess, albuminous deposits; deposits of bony matter, ossification of arteries; hydatids in the ventricles, in the plexus choroides, and even in the brain; alterations of colour; cavities; unusual size of concretions in the pineal gland; diminished size of the ventricles, or their distension by fluid, &c. A case of suicide is referred to, related by Auenbrugger, in which there had been long-continued headach, and a fissure was found in the middle of the pons Varolii. Meckel's experiments on the diminished weight of the brain in lunatics, and on its comparative weight after various diseases, and in man and animals, and of the brain and cerebellum, are referred to; but so many accidental circumstances may influence the results of such experiments, that it does not seem necessary to dwell upon them. Cabanis was of opinion that there was a greater quantity of phosphorus in the brains of lunatics, and especially of suicides, than in persons of sound intellect.

Bloody serum has sometimes been effused in the canal of the dorsal spine, and softening of the extremity of the lumbar cord has been observed.

The sections on the lesions found in the thorax and abdomen of suicides are as interesting in relation to insanity in general as to suicide; and we might extract from Dr. A.'s book many cases of suicide in which the previous symptoms being chiefly referrible to the brain, the only palpable lesions were found in the abdomen or the chest; cases subversive of the vain doctrine that, wherever there is functional disorder, there is and must be physical lesion of the disordered organ.

Every physiological student is familiar with the sympathies existing between the brain and the organs of circulation and respiration, and with the important relations of the latter to the integrity of the cerebrum. Some authors have gone so far as to consider impeded respiration a frequent cause of melancholia, and of the suicidal kind; and every practitioner has occasionally observed cerebral excitement connected with diseases of the heart. Both melancholia and mental excitement are now and then seen palpably to induce or hasten the progress of phthisis. Lesions of the lungs are, perhaps, among the most common morbid appearances in the bodies of lunatics; and Esquirol states that one-fourth of the melancholic die of phthisis pulmonalis. Of the insane who die at the Salpetrière, Georget has found that more than one-half die phthisical; the disease being always in a chronic form, and often unsuspected before death. The difficulty in these cases is to distinguish collateral events from causes and effects.

A disordered state of the skin, with suppressed perspiration, has often been remarked in connexion with melancholia; and Dr. Arntzenius speaks of it under the head of Affections of the Circulation. The actual relation of diseases of the heart and long-continued mental disturbance is not more easy to be determined than that of some mental and pulmonary disorders; their frequent coexistence is well known. It appears that, in many suicides, the heart has been found diseased, enlarged, or contracted, of unusual dryness, misplaced, or the pericardium adherent.

Between the abdominal viscera and the brain, the sympathies have long been acknowledged. The figurative language of all countries conveys the assurance of this conviction having long prevailed. If, with reference to the chest, we have the expressions "suffocated," "breathless," "heart-broken," indicative of various degrees of emotion, the stomach, the liver, the spleen, and the bowels have been equally commemorated in the greater number of languages, both by the vulgar and the learned. Daily experience teaches the effects on the functions of the brain of various stimulants and sedatives received into the stomach; food, wine, opium, &c.; and few observers of their own economy are ignorant of the mental depression attendant on overloaded and sluggish intestines. The effects of injuries of the head upon the stomach, and the relief of the brain often ensuing on the administration of emetics, are things long observed and commonly known; as well as the various shades of cerebral irritation, stupor, sensorial affection, convulsion, and disease arising in intestinal sources.

Loss or excess of appetite, heat of stomach, nausea, vomiting, and obstinate constipation, are circumstances observed in the majority of lunatics; so that the ancients, and even the moderns down to Boerhaave, have been prone, as observed by the author, to consider disturbances of the bile as the general cause of all mental disorders. Dissections of melancholy patients and of suicides have shewn the size of the stomach increased or diminished, inflamed or scirrhous. The small intestines, however, are more frequently the subject of inflammation than the stomach, and especially, it is said, the jejunum. The intestines are not unfrequently found infarcted with fæces; and even the appendix of the cæcum. Displacement and contraction of the larger intestines are not unfrequent. Esquirol found displacement of the colon in several species of insanity, but especially in the melancholic; the transverse portion having become oblique, or even perpendicular; the left portion tending towards or descending behind the pubes, or even the middle portion fallen into the hypogastrium. These displacements were found in 33 bodies out of 168 subjects of melancholia examined. Esquirol considers the epigastric pains and constipation from which lunatics suffer as arising from this cause; for the removal of which he recommends the tartar emetic and various kinds of exercise. Elvert pointed out this circumstance, in his work "Ueber den Selbstmord," published in 1794; and Dr. Arntzenius alludes to a case reported by Pruys, (Rott. 1828,) in which similar displacement of the colon was found in a boy who had been fatuous from birth, and died of quotidian intermittent, with violent delirium. Cases of displacement are also referred to, as being mentioned by Desgenettes, Ballin, Henze (*Hufel. Journal*, 1822,) and Falret. Contraction of the colon has been previously noticed by several authors as of frequent occurrence in maniacal subjects; but this appearance is not uncommon in patients dying of other diseases. Dr. Schlegel relates the cases of two females, who, in consequence of the pains which they complained of in their bowels, inflicted extensive wounds with knives in the situation of the pain. In one, during a fit of melancholy, a cut was made through the abdominal walls, six inches in length. The intestines escaped, but were replaced, and the patient recovered. She afterwards excused the act, on account of a "painful sensation in the belly, from which she wished to relieve herself; a consequence of suppressed menstruation without pregnancy." The subject of the other case lost her life, from the wound which she inflicted with the view of relieving a constant abdominal pain.

The liver, continues Dr. A., is far less frequently found diseased in suicides, and even in maniacs in general, than the commonly entertained opinion among the older physicians would render probable. In 168 examinations of patients affected with melancholia, Esquirol only found two instances of organic lesions of this viscus; and Pinel only found five in 259 examinations of the bodies of lunatics. But considerable functional disorder of the liver may exist during life, and leave no organic trace. Worms (lumbrici) have sometimes been found in the biliary ducts, as well as in the intestines.

Bichat was of opinion that the pancreas was often affected in hypochondriasis, and it has sometimes been found diseased in suicides. Dr. Arntzenius quotes Heister's relation of two cases, in one of which the pancreas was scirrhous, and in the other enlarged and gorged with dark-coloured blood: in both cases there was vitiated bile and disease of the gall-bladder and excretory duct. A case of a suicide, in which a large lumbricus teres was found in the pancreas, is quoted from Mauchart; but in the same case there was induration (completam concretionem) of the lungs, liver, and spleen, and other morbid appearances.

Dr. Arntzenius alludes to the "English malady," in proof of the spleen being often implicated as a cause of melancholy and of suicide; but its lesions are not, it would appear, very frequent. Renal calculi are mentioned by Lorry as occurring in melancholy subjects; and a case is quoted from in which the scrotum was full of calculous concretions. In women, the ovaria are sometimes diseased.

Researches into the morbid anatomy of suicides failing to shew more than the intimate connexion existing between such cases and cases of general insanity, and between the moral affection and corporeal disease, and leading to no satisfactory conclusion as to the primitive cause, the author relates several cases of suicide, with dissections, to afford an opportunity of comparing the moral causes and the organic lesions, before giving the opinions of different authors respecting the seat and nature of this unhappy disease. The chapter entitled "Historiæ et Autopsiæ" contains an account of twenty-seven cases, original or extracted from various authors. Many of these are very interesting and valuable, both in a psychological and pathological point of view; but our limits will only allow us to extract one as a specimen: it is reported by Dr. A. himself, and is for nothing more remarkable than for shewing the fallacies of diagnosis.

*Case* 25. A man, forty-six years of age, of robust constitution and very active habits, temperate in diet, and of a happy disposition, experienced a loss of property which, although not very considerable, strongly affected his mind and his feelings of honour. Before that time, after the strongest exercise, his breathing had never been affected; but after this mental emotion he suddenly became troubled with difficult respiration, a sense of pressure in the right hypochondrium, a dry cough, pains in the shoulders, and depression of mind. No measures relieved him. For nine months he continued to get worse; palpitation, loss of sleep, and fear of death supervened; his temper became morose, he drank spirits, he grew fat, and became subject to hæmorrhoids. At length, on returning one evening from a convivial party, he complained of extreme heat and anxiety, walked up and down his room, would not go to bed, and begged the by-standers to take care of him. After some hours he became more tranquil, and as if meditative, and suddenly threw himself out of the window into the street, by which he suffered compound fracture of the thigh and other injuries. Copious bleeding and appropriate treatment restored his sensibility, but he had no recollection of what had taken place. Symptoms of commotion of the brain followed, but the previous symptoms of diseased heart entirely disappeared. In seven weeks he resumed the management of his affairs, but appeared more taciturn, anxious, and timid, than he had formerly been. By degrees, and in the course of several months, his former symptoms returned, and he became subject to violent accessions of anger. He now also complained of deep-seated, continual, and circumscribed pain in the middle of the occiput, of a feeling of weight in his head, of specks and sparks before his eyes, and of frequent vomiting of bile. Organic disease in the head was supposed to exist, and trepanning recommended but not performed. Nothing afforded him the least relief. He often sate for hours in one place, inattentive to all that passed around him; and often declared that he should go mad. Furious symptoms succeeded, with a desire to murder those near him, and he was removed to a lunatic asylum, from whence in seven months he returned home, looking well, and appearing to be of sound mind, but depressed, silent, averse to society, and restless as if from severe pain. He complained of cephalalgia, vertigo, and anxiety about the præcordia; but he continued for two months to transact business, seemed sane, was careful in his diet, and went about without restraint. About that time his wife became ill, and he removed to a separate bed-room, that out of the window of which he had formerly thrown himself. On the very first night of being there, he fastened the door on the inside, and when it was forced open, he was found to have hanged himself. It appeared that he had been making preparations for this act for some days.

We may suppose the feeling of interest with which an examination of the body would be made in this case. The first result was, that there was no morbid appearance of any kind in the brain or in the spinal cord. The heart was very much enlarged, its base projecting near the sternum, its apex uniform and reflected backward upon the convex surface of the diaphragm; the muscular substance of both ventricles hard and thickened, their cavities very small in proportion, and containing scarcely two drachms of red fluid blood, resembling syrup; the valves and columnæ carneæ thickened and rigid. The external superficies of each auricle, although they contained only a small quantity of red glutinous blood, nearly exceeding by two-thirds the superficies of the ventricles: the cavity of the right auricle thrice the size of that of the right ventricle, and its parietes very thin, and resembling the parietes of the urinary bladder after maceration in water and forcible distension of the muscular coat; so that interstices of from half a line to two lines were observed between the muscular fibres, which were only contained by the investing pericardium, itself in many places as thin as the pia mater, and pellucid. The left ventricle, although larger than natural, in other respects normal. The right lung everywhere adherent to the pleura and pericardium, dense, and gorged with blood; little crepitation on division of its substance, and frothy blood with pus flowing from the divided surfaces. The left lung healthy, but full of blood. The liver of natural size, its upper surface so convex as to carry the diaphragm as high as the middle of the chest; its substance harder than natural; its colour a deep violet. The spleen of enormous magnitude, weighing twenty-one medicinal pounds, and very soft: the vasa brevia and all the abdominal veins much dilated. Varicose vessels on the parietes of the stomach at the great curvature. The ileum exhibited a diverticulum resembling the vermiform process of the cæcum coli, but of three times the thickness, and three inches and a half longer. The omentum and mesentery much loaded with fat; the plexus solaris of a rose-colour and harder than usual.

The reader of all the cases so carefully detailed by Dr. Arntzenius will not, we fear, find himself much advanced towards a knowledge of the seat and nature of suicide; but he will learn that in every case of suicide dissection would probably reveal actual disease of the brain, or so much disease of other organs as might reasonably be supposed to have violently disturbed the cerebral functions.

It is in consequence of the omission of such examination that the remarks made by Dr. Schlegel in the cases of suicide arising from physical, and those from non-physical causes, are not available in a scientific investigation. He speaks of cases of suicide consequent on disease or organic disorder of the body, and of those arising from fear of punishment, deficiency of food, gambling, unhappiness in wedded life, poverty, &c. The former he terms physical, the latter non-physical. But the question still remains to be answered, how far these latter causes have or have not been productive of certain tangible or intangible physical changes, on which the suicide is consequent, and whether they must not therefore be classed with the former? Dr. Schlegel does not appear to participate in the feelings of compassion with which suicides are commonly regarded. He speaks of the act as being " mostly spontaneous, undertaken after free deliberation, and accomplished during perfect consciousness, and one which must be regarded as among the greatest and most abhorred crimes for which an individual can be responsible, and which the cowardly and vicious perpetrator can commit but once." This is a bold judgment to pass, and one in which we cannot coincide. The impulse under which a human being is compelled to take away that which is so valuable as life, or, on the other hand, the state of mind in which he regards it as so valueless as no longer to be worthy of preservation, are conditions on which we can probably pass no correct judgment, and where silence would be the wiser course.

In cases where a tendency to suicide is declared, (and it is not unfrequently avowed by those who show that they were sincere in the avowal,) the practitioner is not left wholly without indications of cure or mitigation of the mental malady. The important lesson taught by the details communicated by Dr. Arntzenius is one which disposes us still less to coincide with the German author in his condemnation of suicides. We close his book by a conviction that, upon all unhappy beings who have died by their own hands, the charitable sentence should be that they were insane. The appearances after death are identical with those commonly found in cases of mania and melancholy; and the previous history of the patient will probably in every case shew a signal departure from ordinary habits previous to the event. The act of suicide is not the effect of specific morbid lesion; it may ensue on any disturbance of the brain, primary or secondary; and the introduction of the particular idea of self-destruction may often be unaccounted for by the nature of existing disease, and only capable of explanation by a more intimate knowledge of the habitual trains of thought of the individual than we can often in any way obtain possession of. The same difficulty, and to the same extent, exists in other cases of monomania; cases in which a single idea, originating in mystery, takes dominion over the whole mind. This may even be the case when there is not only no visible disease of the brain, but no notable affection of any other organ. The healthy condition of the brain may have been suspended in consequence of a violent moral impression; its energy may be impaired and its substance apparently unchanged.

Dr. Arntzenius quotes Dr. Gall's opinion, that suicide arises from too great a predominance of the organ of cautiousness; but later phrenologists, and particularly Mr. Combe, with more correctness add that with this predominance a deficient development of Hope, and a large Destructiveness, must be combined. The cause of phrenology gains little, we fear, from these attempts to be at once brief and precise. The diseased ideality, the morbid conscientiousness must often be taken into consideration; and peculiar modes and degrees of action of other organs.

---

